# Focusing In on the Complex Genetics of Myopia

**DOI:** 10.1371/journal.pgen.1003442

**Published:** 2013-04-04

**Authors:** Robert Wojciechowski, Pirro G. Hysi

**Affiliations:** 1 Department of Epidemiology, Johns Hopkins Bloomberg School of Public Health, Baltimore, Maryland, United States of America; 2 Statistical Genetics Section, Inherited Disease Research Branch, National Human Genome Research Institute (NIH), Baltimore, Maryland, United States of America; 3 Department of Twin Research and Genetic Epidemiology, King's College London School of Medicine, London, United Kingdom; Georgia Institute of Technology, United States of America

## Refractive Errors Are Important Public Health Problems

Myopia (i.e., nearsightedness) is rapidly becoming a significant public health problem. The incidence of myopia has been increasing for at least three decades [1], [2], and 2.5 billion people will be affected by myopia by the year 2020 [3]. In some East Asian countries, the prevalence of myopia now exceeds 70% among teenagers and young adults [2], [4], [5]. Even though ocular refraction (the quantitative trait underlying myopia) is highly heritable [6], the recent change in the incidence of myopia is obviously not the result of short-term shifts in the genetic makeup of the population. Instead, secular trends in environmental and behavioral factors are thought to be driving the myopia “epidemic” throughout the world.

## The Etiology of Myopia: A Historical Dilemma

**Figure 1:**
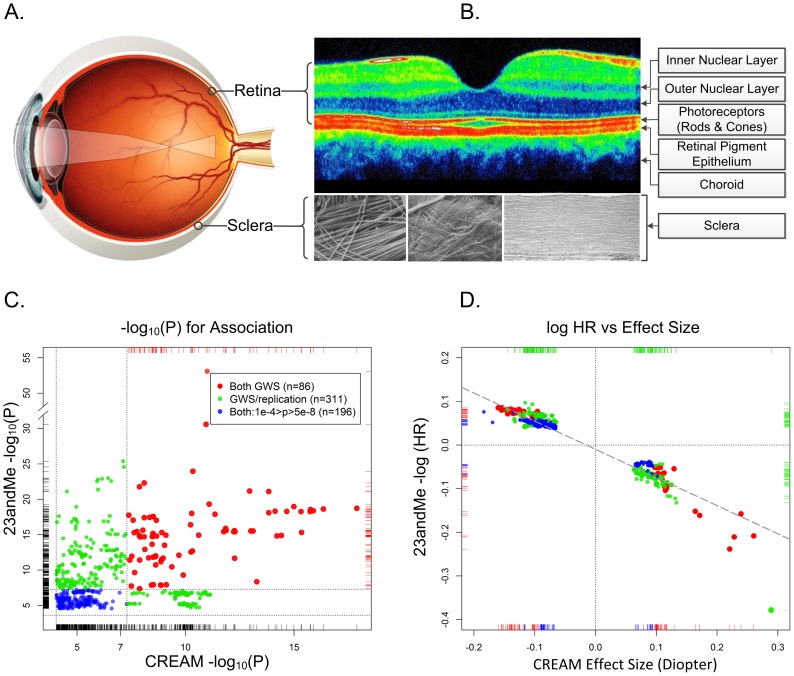
The physical, biological, and genetic basis of myopia. Panel A. The optical basis of myopia. Parallel lights from distantly viewed object focus anterior to the retinal plane, causing blurred distance vision. Although myopia can result from alterations in the optical system (the cornea and lens), myopia is almost always the result of excessive growth of the posterior segment of the eye. This growth occurs through active remodeling of the fibrous sclera. Panel B. Optical coherence tomography cross section of a normal posterior pole. The depression in the middle is the fovea, where the concentration of photoreceptors (and by extension the visual acuity) is greatest. Only four of the ten layers of the neural retina are pointed out. In refractive regulation, visually induced signals are generated by the photoreceptors and processed by other retinal cells (notably, by the amacrine cells, which release neuromodulators and neurotransmitters (including dopamine)). These retinal signals must traverse the retinal pigment epithelium and the highly vascularized choroid in order to regulate eye growth through active remodeling of scleral ECM. The sclera is composed mainly of collagens, but also contains active fibroblasts and normal constituents of ECM (proteoglycans, *MMP*s, *TIMP*s, etc.). The composition of the sclera with its organized structure of collagen fibrils are shown under three different magnifications. Panel C. Statistical significance and effect sizes for 23andMe and CREAM studies. Statistical significance of overlapping SNPs with association p-values <1e-04 in both datasets are plotted. Red dots show SNPs that were genomewide significant (GWS; p<5e-08, −log_10_(p)>7.3) in both studies. Green dots represent SNPs that were genomewide significant in one study and fulfilled our statistical criterion for replication in the other study. Blue dots show SNPs whose p-values for association were between p = 10e-4 and 5e-8 in both datasets. The replication thresholds were the Bonferroni-corrected p-values for a family-wise error rate of 0.05: since the CREAM dataset contained 544 (nonindependent) SNPs that were genomewide significant in 23andMe, the Bonferroni-adjusted p-value threshold for replication was 0.05/544 = 9.2e-05 (or −log_10_(p) = 4.037). Similarly, there were 308 genomewide significant SNPs in CREAM that were also in the 23andMe dataset presented in Table S2 of Kiefer et al. The replication threshold was therefore 0.05/308 = 2.4e-04 (−log_10_(p) = 3.615). Dotted horizontal and vertical lines show the thresholds for genomewide significance and replication. Panel D. Natural logarithm of hazard ratios for myopia in 23andMe vs. effect size (beta coefficient) in CREAM. Both analyses assumed an additive effect of the representative allele on the scale used in the analyses. Color coding is identical to the left panel. The dashed diagonal line shows the linear regression of the 23andMe log hazard ratios on CREAM effect sizes. All SNPs are concordant as to the expected direction of their effects (i.e., positive HRs for myopia would be expected to increase the severity of myopia and negative HRs would be expected to decrease the degree of myopia). Dotted lines show the origins where the effects are null.

The etiology of myopia has been the subject of conjecture for centuries. Johannes Kepler, who published the first comprehensive treatise on the optics of the eye and myopia in 1604 [7], attributed his own nearsightedness to intense study. Ever since, scientists have engaged in a heated *nature versus nurture* debate on the causes of myopia. Inherited and congenital forms of myopia have been documented since at least 1906 [8], [9]. Not until the late 1970s did experiments conclusively show that myopia could be induced during postnatal development by altering the visual experience of animal models through form deprivation or optical defocus [10]–[12]. Subsequent human and animal studies have led to the development of a robust model for refractive control during infancy, dubbed the *emmetropization* process. Although the details of this mechanism are still emerging, its effect is to maintain homeostasis by adjusting eye growth so that the plane of the retina coincides with the focal point of the eye's optical system, much like a camera lens focuses an image on its sensor. In this paradigm, altered visual stimuli (e.g., optically blurred images) initiate a signaling cascade that originates locally in the sensory retina, traverses the retinal pigment epithelium and the vascular choroid, and ultimately regulates eye growth via active remodeling of the sclera—the rigid white connective tissue that forms the outer layer of the eye globe (Figure 1, panels A and B). Alterations within this sequence of biochemical events may disrupt this finely tuned mechanism and lead to refractive errors, resulting in blurred vision. Hence, any gene that plays a role in this complex signaling pathway may contain susceptibility variants for myopia.

## Large GWAS Discovers Numerous Myopia Susceptibility Variants

In a recent issue of *PLOS Genetics*, Amy K. Kiefer and a group from 23andMe, Inc. [13] reported the results of the largest GWAS (n = 45,771) of a refractive phenotype conducted to date. In addition to confirming previously reported loci [14], [15], they discovered 20 novel loci associated with age of onset of myopia in a European-derived population. Their Cox proportional hazards survival analysis approach proved quite powerful and was ideal for the nature of the data collected: given that the vast majority of myopes develop the condition by early adulthood, ascertainment is effectively complete by age 30. Their findings provide unprecedented insight into the genetics of human myopia and may very well transform our understanding of this perplexing condition. Specifically, the authors identified novel loci containing genes involved in a variety of mechanisms putatively related to visual perception and refractive control, including extracellular matrix (ECM) remodeling, the visual cycle, neuronal development, eye and body growth, retinal cell development, and neural signaling. Their data substantiate the assumption that myopia (more precisely: the myopias) is a complex, multifactorial, and genetically heterogeneous condition. Though their precise roles in refraction regulation are currently unknown, many of these genes' molecular functions and biological processes can be placed within the broader biological context of a retina-to-sclera signaling cascade, similar to the one that maintains optical homeostasis during infancy.

Kiefer et al.'s study was not without some limitations. Most notably, their use of questionnaire data, not validated against medical records or eye exams, likely resulted in substantial misclassification. Moreover, age of onset may not be relevant to the severity of myopia, which is ultimately the main outcome of interest for most clinicians and scientists.

As with most complex diseases, Kiefer et al. found no smoking gun (myopia susceptibility variants did not follow the vision genetics precedent of *CFH* as the statistical geneticist's equivalent of a slam dunk). Despite highly statistically significant associations, no variant was estimated to have a strong effect on myopia onset: the hazard ratios among the most highly significant SNPs ranged from 0.79 (rs12193446; *LAMA2*) to 1.15 (rs1381566; *LRRC4C*). The more common associated polymorphisms had quite modest effects, conferring relative hazard differences of less than 10%. These results suggest that we are pushing the boundaries of the power of traditional GWAS to detect myopia susceptibility genes with reasonably sized studies (although the effect estimates in this study are likely biased toward the null given the misclassification rates expected from questionnaire data). If common variants of large effect did exist, however, Kiefer et al. would surely have found them.

## The Three Laws of GWAS: Replication, Replication, Replication

Conventional (i.e., conformist) dogma, in a world of infinitesimal p-values, states that GWAS results are unreliable (minus confirmation through “replication”) no matter the size or statistical power of the “discovery” sample. Kiefer et al. used a validation set of over 8,323 participants who reported on their use of corrective eyewear for myopia before age ten. Using a quite liberal threshold, they statistically replicated ten of their novel loci.

## Independent Studies Unequivocally Confirm Their Association Signals

Many readers may be skeptical of replication p-values on the order of 0.01, especially given multiple testing issues. Kiefer et al.'s replication cohort was, to be sure, underpowered to replicate small effects. Nonetheless, their study was, in fact, remarkably successful as revealed by a large independent study: the Consortium for Refractive Error and Myopia (CREAM), of which we are members. CREAM concurrently conducted a meta-analysis of GWAS comprised of 27 studies (n = 37,382) of adults of European descent and five Asian cohorts (n = 8,376) [16]. The study-specific mean ages ranged from 31.4 to 79.9 years, and the average ages in 26 of these studies were >45 years. Unlike the questionnaire data used by Kiefer et al., CREAM phenotypes consisted of direct objective measurements of the quantitative trait, ocular refraction, measured using autorefractometry. Astonishingly, this independent meta-analysis codiscovered genomewide significant association signals (p<5e-08) at 11 of 20 of Kiefer et al.'s “novel” loci (Figure 1, panel C). An additional five loci met our conservative threshold for statistically significant replication based on a Bonferroni correction. In total, 16 of the 20 novel loci identified by Kiefer et al. were confirmed by CREAM; and of the 22 loci discovered by the CREAM analyses, 14 were replicated by 23andMe (Figure 1, panel C). Arguably, nine additional regions could have been added to the list of replicated loci because both studies contained highly significant signals at these loci, albeit at different SNPs.

Perhaps even more surprising was the fact that, although the demographics, analyses, and measurement scales differed markedly between the respective studies, there was a linear relationship between the *effect sizes* of the statistically significant SNPs common to both studies (Figure 1, panel D). It is quite striking that locus-specific hazard ratios for myopia age of onset (all occurring before age 30) would have predictable, though often small, consequences on the degree of refractive error throughout middle age, and well into the golden years. These relative effects are evident despite substantial changes in refractive error throughout life, in addition to the cumulative influence of a plethora of environmental and behavioral risk factors on refraction.

## What's Next?

There is no doubt that the findings published by Kiefer et al. will contribute significantly to our understanding of the processes by which common forms of myopia develop. Their results were strongly (in many cases unequivocally) validated by a large consortium of refractive error GWAS. Survival analysis of population-based samples, however, may not be adequate to detect variants for rare myopia subtypes, such as Mendelian forms of high myopia. For these cases, sequencing of highly ascertained pedigrees should offer better odds of identifying rare causal variants.

Many of the genes identified by the 23andMe and CREAM groups can be linked to biologically plausible pathways for refractive error control. The eye-growth signaling pathway models developed by biologists had previously defined broad constraints on the mechanisms by which ocular development should be controlled by visual inputs. However, scant few *genetic* animal models for myopia have been developed [17], [18]. Moreover, to our knowledge, none of the refractive error loci identified in the 23andMe and CREAM GWAS have been implicated in visually induced experimental myopia. Hence, the exact mechanisms through which these genes act to regulate ocular refraction are unknown, and current animal models may be inadequate to hypothesize mechanisms for later-onset myopia in humans. The scientific focus will now go back to biology, bioinformatics, and computational biology in an attempt to untangle the effects of these gene networks. Both the 23andMe and CREAM groups have shown that there is no substitute for large sample sizes, and we expect the trend of accumulating ever-larger datasets to continue. Fortunately, refractive error data are easily obtained noninvasively, and Kiefer et al. have shown that age of onset of myopia can be powerful in detecting the signal of refractive error variants.

Future studies should not, however, neglect the large effect of extrinsic factors on refractive error. A significant body of work has linked refractive errors with a number of environmental, ecological, and behavioral risk factors (summarized in, e.g., [6], [19]). In fact, societies in which these putative risk factors are uncommon have low prevalences of myopia [20]–[23]. This suggests that, despite high heritability, exposure to deleterious environmental influences (i.e., reading and near work) may be essential for refractive variation to be manifested. Hence, a clearer picture of myopia and refractive error genetics will require incorporating measures of environmental exposures into our statistical analyses in GWAS. Finally, it is imperative to conduct whole-genome searches in a variety of populations and ethnicities, not only to further validate these results, but to investigate the causes of the wide disparities in the distribution of ocular refraction within, and among, these groups.

## References

[1] Vitale S, Sperduto RD, Ferris FL 3rd (2009) Increased prevalence of myopia in the United States between 1971–1972 and 1999–2004. Arch Ophthalmol 127: 1632–1639. 20008719 10.1001/archophthalmol.2009.303

[2] Lin LL, Shih YF, Hsiao CK, Chen CJ (2004) Prevalence of myopia in Taiwanese schoolchildren: 1983 to 2000. Ann Acad Med Singapore 33: 27–33. 15008558

[3] Kempen JH, Mitchell P, Lee KE, Tielsch JM, Broman AT, et al (2004) The prevalence of refractive errors among adults in the United States, Western Europe, and Australia. Arch Ophthalmol 122: 495–505. 15078666 10.1001/archopht.122.4.495

[4] Wu HM, Seet B, Yap EP, Saw SM, Lim TH, et al (2001) Does education explain ethnic differences in myopia prevalence? A population-based study of young adult males in Singapore. Optom Vis Sci 78: 234–239. 11349931 10.1097/00006324-200104000-00012

[5] He M, Zeng J, Liu Y, Xu J, Pokharel GP, et al (2004) Refractive error and visual impairment in urban children in southern China. Invest Ophthalmol Vis Sci 45: 793–799. 14985292 10.1167/iovs.03-1051

[6] Wojciechowski R (2011) Nature and nurture: the complex genetics of myopia and refractive error. Clin Genet 79: 301–320. 21155761 10.1111/j.1399-0004.2010.01592.x PMC3058260

[7] Kepler J (1604) Ad Vitellionem paralipomena, quibus astronomiae pars optica traditur… Tractatum luculentum de modo visionis, & humorum oculi usu, contra opticos & anatomicos. Frankfurt: Claudius Marnius & heirs of Joannes Aubrius.

[8] Worth C (1906) Hereditary influence in myopia. Trans Opthal Soc U K 26: 141–144.

[9] Wold KC (1949) Hereditary myopia. Arch Ophthalmol 42: 225–237. 10.1001/archopht.1949.00900050231001 18141056

[10] Sherman SM, Norton TT, Casagrande VA (1977) Myopia in the lid-sutured tree shrew (Tupaia glis). Brain Res 124: 154–157. 843938 10.1016/0006-8993(77)90872-1

[11] Wallman J, Turkel J, Trachtman J (1978) Extreme myopia produced by modest change in early visual experience. Science 201: 1249–1251. 694514 10.1126/science.694514

[12] Wiesel TN, Raviola E (1977) Myopia and eye enlargement after neonatal lid fusion in monkeys. Nature 266: 66–68. 402582 10.1038/266066a0

[13] Kiefer AK, Tung JY, Do CB, Hinds DA, Mountain JL, et al (2013) Genome-wide analysis points to roles for extracellular matrix remodeling, the visual cycle, and neuronal development in myopia. PLoS Genet 9 (2) e1003299 doi:10.1371/journal.pgen.1003299. 23468642 10.1371/journal.pgen.1003299 PMC3585144

[14] Hysi PG, Young TL, Mackey DA, Andrew T, Fernandez-Medarde A, et al (2010) A genome-wide association study for myopia and refractive error identifies a susceptibility locus at 15q25. Nat Genet 42: 902–905. 20835236 10.1038/ng.664 PMC4115148

[15] Solouki AM, Verhoeven VJ, van Duijn CM, Verkerk AJ, Ikram MK, et al (2010) A genome-wide association study identifies a susceptibility locus for refractive errors and myopia at 15q14. Nat Genet 42: 897–901. 20835239 10.1038/ng.663 PMC4115149

[16] Verhoeven V, Hysi P, Wojciechowski R, Fan Q, Guggenheim J, et al (2013) Genome-wide meta-analyses of multiancestry cohorts identify multiple new susceptibility loci for refractive error and myopia. Nat Genet 45: 314–318. 23396134 10.1038/ng.2554 PMC3740568

[17] Chen YP, Hocking PM, Wang L, Povazay B, Prashar A, et al (2011) Selective breeding for susceptibility to myopia reveals a gene-environment interaction. Invest Ophthalmol Vis Sci 52: 4003–4011. 21436268 10.1167/iovs.10-7044

[18] Troilo D, Li T, Glasser A, Howland HC (1995) Differences in eye growth and the response to visual deprivation in different strains of chicken. Vision Res 35: 1211–1216. 7610582 10.1016/0042-6989(94)00230-j

[19] Morgan I, Rose K (2005) How genetic is school myopia? Prog Retin Eye Res 24: 1–38. 15555525 10.1016/j.preteyeres.2004.06.004

[20] Pokharel GP, Negrel AD, Munoz SR, Ellwein LB (2000) Refractive error study in children: results from Mechi Zone, Nepal. Am J Ophthalmol 129: 436–444. 10764850 10.1016/s0002-9394(99)00453-5

[21] Casson RJ, Kahawita S, Kong A, Muecke J, Sisaleumsak S, et al (2012) Exceptionally low prevalence of refractive error and visual impairment in schoolchildren from Lao People's Democratic Republic. Ophthalmology 119: 2021–2027. 22698982 10.1016/j.ophtha.2012.03.049

[22] Peet JA, Cotch MF, Wojciechowski R, Bailey-Wilson JE, Stambolian D (2007) Heritability and familial aggregation of refractive error in the Old Order Amish. Invest Ophthalmol Vis Sci 48: 4002–4006. 17724179 10.1167/iovs.06-1388 PMC1995233

[23] Young FA, Leary GA, Baldwin WR, West DC, Box RA, et al (1969) The transmission of refractive errors within eskimo families. Am J Optom Arch Am Acad Optom 46: 676–685. 5258732 10.1097/00006324-196909000-00005

